# Characterization of a Novel Conjugative Plasmid in *Edwardsiella piscicida* Strain MS-18-199

**DOI:** 10.3389/fcimb.2019.00404

**Published:** 2019-11-27

**Authors:** Hossam Abdelhamed, Reshma Ramachandran, Ozan Ozdemir, Geoffrey Waldbieser, Mark L. Lawrence

**Affiliations:** ^1^College of Veterinary Medicine, Mississippi State University, Mississippi State, MS, United States; ^2^Warmwater Aquaculture Research Unit, Thad Cochran National Warmwater Aquaculture Center (USDA-ARS), Stoneville, MS, United States

**Keywords:** antimicrobial resistance, mobile genetic element, *Edwardsiella*, aquaculture, genome sequence

## Abstract

*Edwardsiella piscicida* is a pathogenic bacterium responsible for significant losses in important wild and cultured fish species. *E. piscicida* strain MS-18-199 recovered from a diseased hybrid catfish from East Mississippi and showed resistance to florfenicol, chloramphenicol, oxytetracycline, doxycycline, erythromycin, tetracycline, azitromycin, spectinomycin, sulfonamide, and bacitracin. To explore the mechanisms of resistance in *E. piscicida* strain MS-18-199, genomic DNA was extracted and subjected to whole genome sequencing (WGS) using a combination of long (Oxford Nanopore) and short (Illumina) reads. The genome of strain MS-18-199 revealed a novel plasmid named pEPMS-18199. The 117,448 bp plasmid contains several antimicrobial resistance (AMR) elements/genes, including florfenicol efflux pump (*floR*), tetracycline efflux pump (*tetA*), tetracycline repressor protein (*tetR*), sulfonamide resistance (*sul2*), aminoglycoside O-phosphotransferase *aph(6)*-Id (*strB*), and aminoglycoside O-phosphotransferase *aph(3)*-Ib (*strA*). Two genes, *arsA* and *arsD*, that encode protein components related to transport/resistance to arsenic were also found in pEPMS-18199. In addition, pEPMS-18199 carried twelve conjugative transfer genes (*tra*), eight transposases and insertion elements, two plasmid stability proteins, two replication proteins, and three partitioning proteins (*par* system). Results from mobilization and stability experiments revealed that pEPMS-18199 is highly stable in the host cell and could be transferred to *Escherichia coli* and *Edwardsiella ictaluri* by conjugation. To our knowledge, this is the first detection of a multidrug resistance (MDR) conjugative plasmid in *E. piscicida* in the United States. Careful tracking of this plasmid in the aquaculture system is warranted. Knowledge regarding the molecular mechanisms of AMR in aquaculture is important for antimicrobial stewardship.

## Introduction

*Edwardsiella piscicida*, a member of family *Enterobacteriaceae*, is a motile Gram negative and facultative anaerobic bacterium (Ewing et al., 1965). The description of *E. piscicida* (previously identified as *Edwardsiella tarda*) resulted from a reclassification of diverse isolates obtained from diseased fish (Abayneh et al., 2013). Besides its importance as a pathogen of aquatic species, *E. piscicida* has been isolated from a wide range of hosts (including birds, reptiles, and humans) from a broad geographical range and ecological niches (Camus et al., 2016; Buján et al., 2018b). At present, *E. piscicida* is the etiologic agent of edwardsiellosis and is responsible for high mortality rates in several fish species (more than 20 fish species as hosts) (Abayneh et al., 2013; Camus et al., 2016; Loch et al., 2017; Buján et al., 2018b). Common clinical signs associated with edwardsiellosis include exophthalmia, abdominal distension, skin hemorrhages, mild to moderate dermal ulcerations, discolorations on the fish surface, and erratic swimming (Buján et al., 2018b).

Commercial production of channel catfish (*Ictalurus punctatus*) and channel catfish × blue catfish (*I. furcatus*) hybrids dominates aquaculture in the United States accounting for $360 million in 2018, and the majority of production occurs in Mississippi (USDA, 2019). Loss to disease and feed cost are the major factors that influence profitable catfish aquaculture (Wagner et al., 2002). Approximately 45% of inventory losses on catfish farms are attributable to infectious diseases, of which 60% are associated with single or mixed bacterial infections (Hawke and Khoo, 2004). *Edwardsiella* species continue to be the bacterial pathogen of primary concern in catfish aquaculture in the southeastern United States (Hawke et al., 1981, 2013). There is a trend toward increased incidence and prevalence of *E. piscicida* septicemia in US catfish aquaculture (Griffin et al., 2019). The recent reclassification and review of archival data indicated that *E. piscicida* is more problematic in catfish aquaculture than *E. tarda* (Buján et al., 2018b; Griffin et al., 2019). Moreover, recent genotypic analysis of bacterial isolates historically classified as *E. tarda* concluded that many isolates previously classified as *E. tarda* actually belong to the species *E. piscicida* (Reichley et al., 2017; Buján et al., 2018a).

Because no definite vaccine is available, antimicrobial (AM) medicated-feeds are the current available strategy to control the bacterial diseases caused by *E. piscicida* and other fish pathogens (Reichley et al., 2017; Buján et al., 2018b). Terramycin (oxytetracycline), Romet-30 (sulfadimethoxine-ormetoprim), and Aquaflor (florfenicol) are three AM agents approved by the FDA for use in medicated-feed to control bacterial infections in cultured fish (Bowker et al., 2010). Although AM use is highly restricted and strongly regulated in the United States aquaculture, a multidrug resistant (MDR) strain of *E. piscicida* strain MS-18-199 (unpublished data) has been recovered from moribund hybrid catfish submitted to Aquatic Diagnostic Laboratories at College of Veterinary Medicine Mississippi State University (CVM/MSU). The mechanisms that drive resistance in this isolate is unclear. Here, we describe the genetic feature of a large conjugative plasmid identified in *E. piscicida*. Moreover, we evaluated plasmid mobility and stability. Characterizing a plasmid carrying resistance determinants, especially in clinical isolates, will increase our understanding of the flow of resistance.

## Materials and Methods

### Bacterial Isolates and Growth Condition

*Edwardsiella piscicida* strain MS-18-199 was isolated from moribund hybrid catfish clinical case presented to the Fish Diagnostic Laboratory at CVM/MSU. The diseased fish exhibited large ulceration on the dorsal head with bone erosion and scant yellow ascites. The internal examination of catfish revealed moderate granulomatous hepatitis. The initial presumptive diagnosis based on clinical signs was *E. piscicida* infection. Pure colonies with identical morphology were isolated from posterior kidney on Mueller Hinton agar (Becton, Dickinson and Company, Franklin Lakes, NJ, USA) supplemented with 5% defibrinated sheep blood (HemoStat Laboratories, Dixon, CA, USA) at 30°C. A stock culture was stored at −80°C in 20% glycerol. The isolate was confirmed phenotypically as *E. piscicida* infection. The API 20E profile of *E. piscicida* strain MS-18-199 is present in Supplementary Table 1. *Escherichia coli* J53Az^r^ (resistant to sodium azide) (Martinez-Martinez et al., 1998), *E. ictaluri* 93-146 with pAK*gfplux1* (ampicillin resistant) (Karsi and Lawrence, 2007), and *Aeromonas hydrophila* ML09-119 (ampicillin resistant) (Tekedar et al., 2013b) were used as a recipient for conjugation. *Edwardsiella* species were cultured in brain heart infusion (BHI) media (Difco, Sparks, MD, USA) and incubated at 30°C with orbital shaking (250 rpm). *E. coli* and *A. hydrophila* strains were cultured in Luria–Bertani (LB) agar and broth (Becton Dickinson) at 30°C and 37°C, respectively.

### Antimicrobial Susceptibility

The susceptibilities of *E. piscicida* strain MS-18-199 to 23 AMs were determined using Kirby-Bauer disk diffusion assays according to CDC guidelines, as described by the Clinical and Laboratory Standards Institute (CLSI) (CLSI, 2010). The tested AMs included: florfenicol FFC30 (30 μg); doxycycline D30 (30 μg); erythromycin E15 (15 μg); oxytetracycline T30 (30 μg); gentamicin GM10 (10 μg); nalidixic acid NA30 (30 μg); chloramphenicol C30 (30 μg); sulfamethoxazole (25 μg); trimethoprim-sulfamethoxazole (1.25 and 23.75 μg); tetracycline TE30 (30 μg); azitromycin AZM15 (15 μg); ceftriaxone CRO30 (30 μg); amoxicillin/clavulanic acid AMC30 (30 μg); ciprofloxacin CIP5 (5 μg); streptomycin S10 (10 μg); spectinomycin SPT100 (100 μg); ampicillin AM10 (10 μg); cefpodoxime CPD10 (10 μg); ceftiofur XNL30 (30 μg); penicillin P10 (10 μg); enrofloxacin ENO 5 (5 μg); bacitracin B10 (10 μg); and novobiocin NB30 (30 μg). Bacterial strains were reported as susceptible, intermediate, or resistant based on inhibition zone size as published in the CLSI standard (CLSI, 2010).

### DNA Isolation

A single colony of *E. piscicida* strain MS-18-199 was grown overnight at 30°C in BHI. Overnight culture was pelleted by centrifugation, and genomic DNA (gDNA) isolated using standard ethanol precipitation following phenol: chloroform: isoamyl alcohol (24:25:1 v/v; AppliChem) treatment, as previously described (Psifidi et al., 2010; Green and Sambrook, 2017). The quality of extracted gDNA was assessed using an Agilent 2100 Bioanalyzer (High sensitivity DNA chip), and a Nanodrop spectrophotometer. Genomic DNA with an ultraviolet absorbance 260/280 of ~ 1.8 and absorbance 260/230 of 2.0–2.2 was used for library preparation.

### Whole Genome Sequence of *E. piscicida* Strain MS-18-199

The genome of *E. piscicida* strain MS-18-199 was sequenced using long and short read technologies. Long reads were produced on a GridION sequencer (Oxford Nanopore Technologies, Oxford, UK) from 400 ng genomic DNA using the RAD004 kit on a v9.4.1 flow cell. The nanopore sequences were filtered to an average quality of 10 (90% accuracy) with 100 bp cropped from each end and minimum length of 700 bp (De Coster et al., 2018) to produce 2.4 Gb of sequence from 185,481 reads ranging from 700 bp to 191 kb (average length 13.4 kb). A genomic DNA library was prepared using the Nextera XT DNA kit (Illumina Inc., San Diego, CA, USA) and 150 bp paired reads were sequenced on the Illumina MiSeq4000 Platform (Novogene Corporation, Sacramento, CA). The Illumina sequence was filtered using Trimmomatic v0.38 (Bolger et al., 2014) using the parameters “LEADING:30 TRAILING:30 SLIDINGWINDOW:4:30 MINLEN:50” to produce 1.6 Gb sequence in 5.5 M paired reads.

### Genome Assembly

The nanopore sequence was assembled *de novo* [Canu v1.7; Koren et al., 2017] into two contigs of 3.9 Mb and 230 kb. The linear contigs were manually trimmed at the sequence overlap within the putative circular genome and plasmid, then sequence from the 3′ end of each contig (1 Mb and 100 kb, respectively) was cut and moved to the 5′ end of each respective contig. The filtered nanopore reads were mapped to these contigs and visualized using the IGV viewer (Robinson et al., 2011) to validate the sequence contiguity and therefore the circular nature of the genome and plasmid. The consensus sequences were corrected using four iterations of Nanopolish v.10.2 (Loman et al., 2015) using the filtered Nanopore sequences, then corrected with two iterations of Pilon v1.2.2 (Walker et al., 2014) using the filtered Illumina reads.

### Genome Annotation and Function Analysis of Strain MS-18-199

The assembled genome was submitted to the National Center for Biotechnology Information (NCBI) database (http://www.ncbi.nlm.nih.gov/) prokaryotic genome annotation pipeline. Antimicrobial resistance (AMR) genes were automatically annotated as resistance genes at the NCBI. Each annotated resistant protein was searched against NCBI database using the best match from a blastn search (NCBI BLAST v2.2.27+). Further confirmation was performed by blast search against the Antibiotic Resistance Database (ARDB) (http://ardb.cbcb.umd.edu/) (Balabanov et al., 2012) and Comprehensive Antibiotic Resistance Database (CARD) (https://card.mcmaster.ca/) (McArthur et al., 2013). The NCBI BLAST results were used to confirm the annotation of insertion sequence, replication, and conjugative proteins. Gene function was predicted using the Gene Rapid Annotation Subsystem Technology (RAST) server (http://rast.nmpdr.org/) (Overbeek et al., 2014).

### Conjugation Experiment

Plasmid mobility was evaluated by conjugation experiments on filter paper placed on agar plates as previously described (Abdelhamed et al., 2018a). *E. piscicida* strain MS-18-199 was used as a donor strain, and *E. coli* J53, *E. ictaluri*, and *A. hydrophila* ML09-119 were used as recipient strains. Briefly, overnight cultures of donor and recipient cells were diluted and incubated at 30°C with shaking until they reached logarithmic phase. Bacteria were harvested by centrifugation at 12,000 rpm, mixed into 1:1 ratio, placed onto filter paper on agar plate, and incubated at 30°C overnight. After mating, bacteria were collected, washed, diluted, and plated on BHI plates containing sodium azide (100 μg/ml) or ampicillin (100 μg/ml) for counter selection, and florfenicol (30 μg/ml) and tetracycline (30 μg/ml) to select for resistance. The transconjugant colonies were counted and the mobilization efficiency was estimated as the number of transconjugants CFU (colony-forming unit) per recipient CFU (Zeng et al., 2015). The resistance phenotypes of the transconjugant colonies were evaluated using the disc diffusion assay as described in the above section. Further confirmation of plasmid carriage in the transconjugant colonies was performed by PCR amplification of florfenicol resistance using FlorR_F (CTGATGGCTCCTTTCGACAT) and FlorR_R (AGACGACTGGCGACTTATCG) primers.

### Plasmid Stability Assays

A stability test of the plasmid in *E. piscicida* strain MS-18-199 was conducted as previously described (Moleres et al., 2015). Briefly, a single colony of *E. piscicida* strain MS-18-199 harboring the plasmid was inoculated in BHI without antibiotic and grown overnight at 30°C. This culture was passaged by serial 1:100 dilutions in 5 ml BHI on consecutive days for up to 20 days. In each subculture step, bacteria were plated on BHI agar, and the proportion of plasmid-containing cells was deduced by replica inoculation of 100 colonies on BHI broth and BHI broth containing florfenicol. The number of colonies carrying the plasmid was calculated as the percentage of florfenicol-resistant colonies in every subculture/total number of colonies replicated each subculture (Moleres et al., 2015).

### Plasmid Copy Number

Plasmid copy numbers in *E. piscicida* strain MS-18-199 were determined using quantitative real-time PCR (qRT-PCR) as previously described (Botts et al., 2017). Briefly, total genomic DNA (gDNA) was extracted from early stationary phase cells using the Genomic DNA Mini Kit (IBI Scientific), as per manufacturer's instructions. The gDNA was quantified then serially diluted two-fold and used as the qRT-PCR template. qRT-PCR was performed in triplicate using SYBR Green Real-time PCR master mix (Roche Diagnostic GmbH, Mannheim, Germany). Chromosomal and plasmid DNA were quantified using primers targeting L-asparaginase 1 gene of *E. piscicida* strain MS-18-199 (locus tag: EVK84_00005; FP: ACCGATACCATGGCGTTTAC; RP: CCAGATAGAGGGCGTTTAACAG), which exists on the chromosome as a single copy, and the single-copy *floR* gene encoded on the plasmid (locus tag: EVK84_18305; FP: TGATCGTGACAACCCGTTTC; RP: GATGAAGGTGAGGAATGACGG).

## Results

### Resistance Phenotypes of *E. piscicida* Strain MS-18-199

The susceptibility pattern of *E. piscicida* strain MS-18-199 to 23 AM agents are shown in Table 1. Strain MS-18-199 was resistant to florfenicol, chloramphenicol, oxytetracycline, doxycycline, erythromycin, tetracycline, azitromycin, spectinomycin, sulfamethoxazole, and bacitracin.

**Table 1:** Antibiotic resistance patterns of *Edwardsiella piscicida* strain MS-18-199.

**Antimicrobial agents**	**Disk content (μg)**	**Diameter of inhibition zone (mm)**	**Sensitivity**
Florfenicol FFC30	30	0	R
Doxycycline D30	30	0	R
Erythromycin E15	15	0	R
Oxytetracycline T30	30	0	R
Gentamicin GM10	10	26	S
Nalidixic acid NA30	30	37	S
Chloramphenicol C30	30	0	R
Sulfamethoxazole	25	0	R
Sulphamethoxazole trimethoprim SXT	25	26	S
Tetracycline TE30	30	0	R
Azitromycin AZM15	15	0	R
Ceftriaxone CRO30	30	35	S
Amoxicillin/clavulanic acid AMC30	30	27	S
Ciprofloxacin CIP5	5	40	S
Streptomycin S10	10	24	S
Spectinomycin SPT100	100	0	R
Ampicillin AM10	10	27	S
Cefpodoxime CPD10	10	23	S
Ceftiofur XNL30	30	35	S
Penicillin P10	10	21	S
Enrofloxacin ENO 5	15	38	S
Bacitracin B10	10	0	R
Novobiocin NB30	30	18	S

### Basic Data of *E. piscicida* Strain MS-18-199 Genome

We conducted whole genome sequencing and assembly to gain further insight on the molecular mechanism of resistance of *E. piscicida* strain MS-18-199. The genome of strain MS-18-199 consisted of one circular chromosome and one circular plasmid, and both sequences have been submitted to GenBank under accession numbers CP035668.1 and CP035669.1, respectively. The 3,905,600 bp genome contained 59.47% GC content and 3,758 genes, including 3,631 coding sequences (CDS), 83 pseudogenes, and 127 RNAs [including 25 rRNAs (9, 8, and 8 for 5S, 16S, and 23S, respectively), 97 tRNAs, and 5 ncRNAs]. The complete genome of *E. piscicida* strain MS-18-199 shared 99.40% average nucleotide identity (ANI) with *Edwardsiella piscicida* C07-08 (Tekedar et al., 2013a), and 99.35% with *Edwardsiella piscicida* isolate S11-285 (Reichley et al., 2016).

### Identification of pEPMS-18199 Plasmid in *E. piscicida* Strain MS-18-199

**Figure 1:**
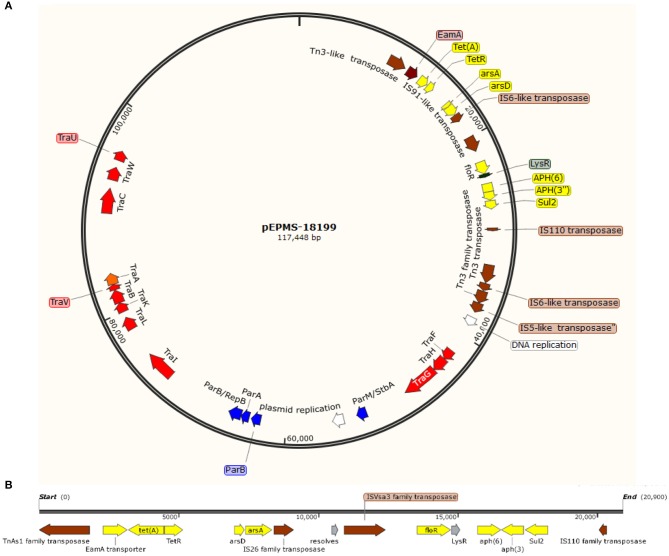
**(A)** Circular genetic map of the pEPMS-18199 plasmid from *Edwardsiella piscicida* strain MS-18-199. The pEPMS-18199 plasmid carries antibiotic resistance (yellow), transposons and insertion sequences (brown), maintenance/stability protein and partitioning proteins (par system) (blue), DNA replication (white), and conjugative transfer genes (red). **(B)** Antimicrobial resistant cassette regions in pEPMS-18199 plasmid.

A circular plasmid was identified from the assembly of long sequencing reads from *E. piscicida* strain MS-18-199. The plasmid pEPMS-18199 is 117,448 bp with an average 47.34% GC content, ~12% lower than the genome. Genome annotation revealed 124 open reading frames (ORFs), of which five were associated with plasmid DNA replication and partition, 12 with DNA transfer, 13 with DNA-restriction and site-specific DNA methylation, eight with family transposase, eight with antimicrobial resistance, and 44 with unknown function “hypothetical protein” (Figure 1A). A BLASTN search revealed pEPMS-18199 was nearly identical (99.97 % identity, ANI 99.94%, and 95% coverage) to 127,046 bp plasmid ET080813 (CP006665.1) isolated from *E. anguillarum* in China (Shao et al., 2015).

Six AMR genes were identified in the pEPMS-18199 sequence, including major facilitator superfamily (MFS) antibiotic efflux pump (*floR*), tetracycline repressor protein (*tetR*), tetracycline resistance protein class A (*tetA*), sulfonamide-resistant dihydropteroate synthase (*sul2*), aminoglycoside O-phosphotransferase *aph(6)*-Id, and aminoglycoside O-phosphotransferase *aph(3*″*)*-Ib (*strA*) (Table 2). Two genes corresponding to proteins predicted to be involved in arsenic resistance were also identified in the pEPMS-18199 sequence; arsenite efflux transporter metallochaperone *arsD* and arsenical pump-driving ATPase *arsA*. All the AMR genes were clustered in one region between 9,052 and 28,696 of pEPMS-18199. This region was flanked upstream and downstream with transposable elements (Figure 1B).

**Table 2:** Predicted AMR elements in pEPMS-18199 plasmid using CARD.

**ARO Term**	**AMR gene family**	**Resistance mechanism**	**% Identity of matching region**	**% Length of reference sequence**
*tetR*	Major facilitator superfamily (MFS) antibiotic efflux pump	Tetracycline antibiotic, glycylcycline	52.74	108.17
*sul2*	Sulfonamide resistant sul	Sulfonamide antibiotic, sulfone antibiotic	100.0	100.00
*tet(A)*	Major facilitator superfamily (MFS) antibiotic efflux pump	Tetracycline antibiotic	79.39	100.76
*floR*	Major facilitator superfamily (MFS) antibiotic efflux pump	Phenicol antibiotic	98.76	100.00
APH(3″)-Ib	APH(3″)	Aminoglycoside antibiotic	99.63	100.00
APH(6)-Id	APH(6)	Aminoglycoside antibiotic	99.64	100.00

The pEPMS-18199 plasmid contains twelve conjugative transfer genes clustered in two regions, designated as Tra1 and Tra2. The Tra1 region is located between sequence coordinates 40,209 and 47,900 and consists of three proteins (TraFHG). The Tra2 region is located between 72,240 and 98,771, and consists of nine proteins (TraI, TraL, TraKBVA, and TraCWU). The pEPMS-18199 plasmid encodes eight transposases and insertion sequences belonging to Tn3-like element TnAs1 family transposase, IS6-like element IS26 family transposase, IS91-like element ISVsa3 family transposase, IS110 family transposase, two Tn3 family transposase, IS6-like element IS26 family transposase, and IS5-like element ISKpn26 family transposase. The plasmid also harbors plasmid stability protein (ParM/StbA family protein) and two plasmid replication proteins. Furthermore, the pEPMS-18199 plasmid encodes additional maintenance proteins including three chromosome partitioning proteins (ParB, ParA, and ParB/RepB family protein).

### General Annotation for pEPMS-18199 Plasmid and *E. piscicida* Strain MS-18-199 Genome

**Figure 2:**
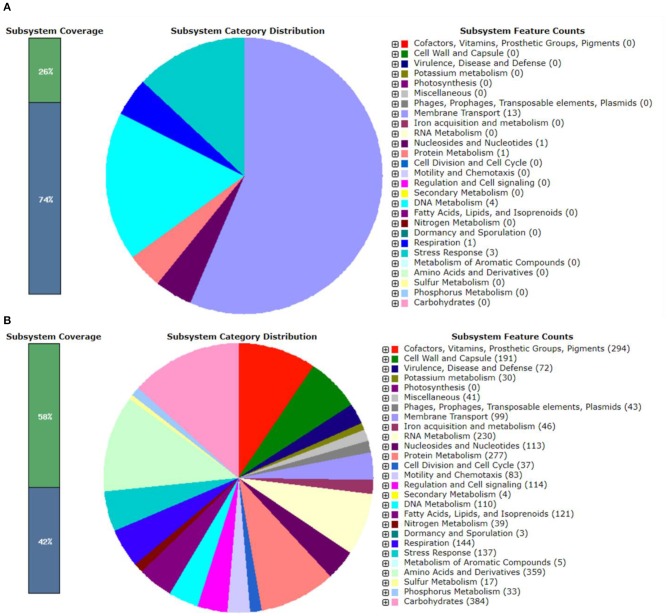
RAST annotation summary of **(A)** pEPMS-18199 plasmid and **(B)** *Edwardsiella piscicida* strain MS-18-199. The RAST annotation robot assigns names and functions to protein-coding genes via their subsystem technology. The green color represents features that are found in RAST subsystem. The blue color represents features not assigned to a subsystem.

The genomic features of pEPMS-18199 plasmid annotated using RAST revealed that pEPMS-18199 contains 13 proteins encoding for membrane transport, one protein encoding for nucleosides and nucleotides, one protein encoding for protein metabolism, four proteins encoding DNA metabolism, one protein encoding respiration, and three proteins encoding stress response (Figure 2A).

The genomic features of chromosome annotated using RAST revealed that *E. piscicida* strain MS-18-199 genome contains 3,551 coding sequences and 497 subsystems (Figure 2B). The most represented subsystem features are cofactors, vitamins, prosthetic croups, pigments (294 genes), cell wall and capsule (191), virulence, disease and defense (72), potassium metabolism (30), phages, prophages, transposable elements, plasmids (43), iron acquisition and metabolism (46), membrane transport (99), RNA metabolism (230), nucleosides and nucleotides (113), protein metabolism (277) fatty acids, lipids, and isoprenoids (121), amino acids and derivatives (359), phosphorus metabolism (33), and carbohydrates (384).

### Mobilization and Stability of the pEPMS-18199 Plasmid

Successful conjugation mating experiments indicated that the pEPMS-18199 plasmid was capable of being transferred from *E. piscicida* to *E. coli* and *E. ictaluri* with an average efficiency of 6.86 × 10^−5^ and 6.13 × 10^−5^ CFU/recipient, respectively. Furthermore, *E. coli* and *E. ictaluri* transconjugants showed the same pattern of resistance as donor strain. Also, PCRs confirmed the presence of plasmid in transconjugants. Conversely, pEPMS-18199 could not be transferred from *E. piscicida* to *A. hydrophila*.

The stability of pEPMS-18199 plasmid was assessed by inoculating and propagating *E. piscicida* in the absence of antibiotic selection. Following 20 serial subcultures without any antibiotic pressure, no colony (among 100 colonies each subculture) had lost the plasmid as indicated by no change in the AMR profiles of the cultures and was further confirmed by PCR.

### Plasmid Copy Number

**Figure 3:**
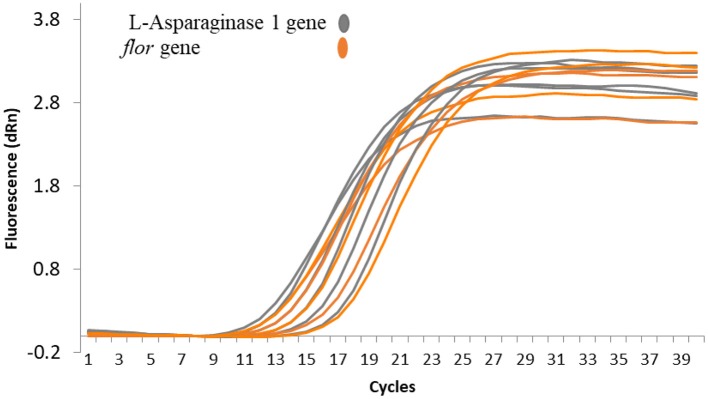
qRT-PCR amplification plots showing the relative plasmid copy number of pEPMS-18199 in *E. piscicida* strain MS-18-199. The cycle threshold (Ct) values obtained for the *floR* gene were similar to the L-asparaginase 1 gene indicating only a single copy of the plasmid is carried by the strain MS-18-199. Orange color represent *floR* gene carried by pEPMS-18199 and gray color represent L-asparaginase 1 gene located in *E. piscicida* chromosome.

Plasmid copy number was determined for pEPMS-18199 in strain MS-18-199 using qRT-PCR by comparing plasmid abundance with that of gDNA. Our result suggests that only a single copy of pEPMS-18199 was carried in strain MS-18-199. Following qRT-PCR, cycle threshold (Ct) values obtained for the *floR* gene representing the pEPMS-18199 were similar to that of the L-asparaginase 1 gene representing the strain MS-18-199 chromosome (Figure 3).

## Discussion

As in other areas of animal production, selection pressures created by antimicrobial (AMs) usage represent a major concern in the aquatic environment. Selection pressure could favor the amplification of resistant bacterial strains and resistance genes. Several studies have documented development of resistant bacteria in and around fish farms in different types of aquaculture production systems (Schmidt et al., 2000; Petersen et al., 2002; Miranda et al., 2003; Alcaide et al., 2005; Furushita et al., 2005; Sapkota et al., 2008). However, little information is available about the molecular basis of resistance. Therefore, the goal of this study was to characterize the genomic feature of the pEPMS-18199 plasmid identified in *E. piscicida* MS-18-199. This in fact will provide an insight into the molecular mechanism of resistance in *E. piscicida* in the aquatic environments. Also, we evaluated the mobility and stability of this plasmid.

The genome sequence of *E. piscicida* strain MS-18-199 demonstrated a MDR-plasmid that is highly similar to a plasmid isolated from *E. anguillarum* ET080813 (CP006665.1) from China (Shao et al., 2015), sharing almost identical backbones (proteins coding for conjugal transfer, replication, stability, and maintenance). The plasmid replication database and Pfam database were used to identify incompatibility group and origin of replication of the pEPMS-18199 plasmid. However, pEPMS-18199 cannot be assigned to any of the historical incompatibility groups. This may be because the incompatibility group includes only plasmids with high nucleotide identity (Shintani et al., 2015).

Six AMR elements were found in the pEPMS-18199 plasmid known to confer resistance to tetracyclines, phenicol compounds, sulfonamides, and aminoglycosides. Resistance of the host strain to these AMs is attributed to the resistance genes located in the pEPMS-18199 plasmid. In previous studies by our group and other investigators, an IncA/C plasmid-mediated carrying of *floR, sul2*, and *tetA* genes was detected in *E. ictaluri* isolated from catfish in the southeastern United States (Welch et al., 2009; Abdelhamed et al., 2018c) as well as *Yersinia ruckeri* and *Aeromonas salmonicida* isolates from salmonids (McIntosh et al., 2008; Lafrentz et al., 2011). In another study, two plasmids harboring *floR, tetA*, and *sul2* resistance genes were reported in a *Plesiomonas shigelloides* strain isolated from catfish in the United States (Abdelhamed et al., 2018b). The similarity of resistance phenotypes and genotypes among *E. piscicida, E. ictaluri*, and *P. shigelloides* isolated from catfish farms suggest the circulation of these resistance elements/genes in catfish ponds and other aquatic environments.

Tetracyclines are among the approved AMs for use on catfish farms. In the present study, the pEPMS-18199 plasmid was found to carry *tetR* family transcriptional repressor and *tetA* tetracycline efflux pump. In fish farm-associated bacteria, tetracycline resistance was found to be mainly due to acquisition of *tet* determinants rather than due to mutation of chromosomal genes (Rhodes et al., 2000). A *tetA* gene has been previously identified in Gram-negative bacteria from fish farms from a number of geographic locations, including in the United States (Miranda et al., 2013). For example, a *tetA* gene located on Inc*K*-plasmid was found in *E. ictaluri* isolates obtained from diseased freshwater catfish (*Pangasianodon hypophthalmus)* in Vietnam (Dung et al., 2009). In another study, the *tetA* gene was located in a smaller plasmid in an *Aeromonas salmonicida* isolate (Schmidt et al., 2001). In general, these findings indicate that *tetA* is main *tet* determinant genes among different plasmids in different fish farm-associated bacteria. However, further explanation on the spread of tetracycline resistance determinants is warranted.

Florfenicol, a chloramphenicol derivative, is effective against a number of important bacterial fish pathogens (Gaunt et al., 2010). In the present study, plasmid pEPMS-18199 was found to carry the *floR* gene, which confers resistance to florfenicol and chloramphenicol. The *floR* gene belongs to a major facilitator superfamily and codes for efflux proteins that export florfenicol out of the cell (Schwarz et al., 2004). RNA methyltransferases and specific hydrolases are other mechanisms of resistance to florfenicol (Schwarz et al., 2004; Long et al., 2006). However, most previous studies on florfenicol-resistant bacterial strains isolated from fish farms reported occurrence of the *floR* gene (Miranda et al., 2013).

In the current study, the *sul2* gene, encoding sulfonamide-resistant dihydropteroate synthase, was also found in the pEPMS-18199 plasmid. The *sul1* and *sul2* genes are predominant forms of *sul* genes in Gram-negative bacteria (Rådström et al., 1991). In the present study, the *sul2* gene found was 816 bases in length and was highly similar to *sul2* present in *Acinetobacter baumannii* and *E. coli*. The *sul1* gene is usually found in plasmids flanked by the insertion element (Rådström et al., 1991). The *sul2* was flanked downstream with IS110 family transposase and flanked upstream with two aminoglycoside resistance genes, *aph(3*″*)-Ib* and *aph(6)-Id*, which confer resistance to streptomycin, a broad spectrum aminoglycoside (van Treeck et al., 1981; Enne et al., 2004). The *aph(3*″*)-Ib* gene is also denominated as *strA* and *aph(6)-Id* as *strB* (Ramirez and Tolmasky, 2010). Furthermore, the cluster of *sul*2*, strB*, and *strA* is commonly found in plasmids isolated from both Gram-positive and Gram-negative bacteria, such as *Aeromonas bestiarum* and *Salmonella* enterica serotype Typhimurium (Daly et al., 2005; Gordon et al., 2008). Interestingly, strain MS-18-199 was found to be susceptible to trimethoprim-sulphonamide combination (SXT), possibly because of the absence of the *dhfr1* gene encoding resistance to trimethoprim in pEPMS-18199 plasmid. The combination of trimethoprim-sulphonamide is approved for use in catfish farms for treating bacterial infections.

The relationship between metal resistance (tolerance) and antibiotic resistance is well recognized (Knapp et al., 2017), but few studies have been reported in fish (McIntosh et al., 2008). The resistance to arsenic is conferred by the presence of *ars* operon, which consists of five genes *arsR, arsD, arsA, arsB*, and *arsC*, located on either the plasmid or chromosome (Qin et al., 2006). In the present study, we demonstrated two genes on pEPMS-18199 that encode proteins that confer resistance to arsenic: *arsD* and *arsA*. The *arsD* is 363bp in length and *arsA* is 969 bp in length. *arsD*/*arsA* regions are flanked downstream with IS6-like element IS26 family transposase. Arsenic and other heavy metals are characterized by long persistent in the environment and may accumulate in soil, water, and sediments from agricultural or industrial practices (Wu et al., 2018).

Plasmid-mediated AMR is often carried through mobile elements, such as transposons (Gordon et al., 2008) and/or integrons (Kummerer, 2004). In the present study, pEPMS-18199 contains eight transposable elements. The *tetA/tetR* gene is associated with a Tn3-like element, TnAs1 family transposase, whereas *floR, aph(3*″*)-Ib, aph(3*″*)-Ib*, and *sul2* are flanked by IS91-like element, ISVsa3 family transposase. Transposons play key roles in horizontal gene transfer and recombination events. Thus, facilitate spread of resistant elements among bacterial isolates and species (Roberts, 1994). In addition, another factor for consideration is that the resistance cassette present in pEPMS-18199 carrying the transposon*s* gene is likely to be able to be mobilized by itself to other bacteria.

There are two Tra regions that encode components of the type IV secretion system in pEPMS-18199. Data from conjugation experiments demonstrated that pEPMS-18199 can mobilize to *E. coli* and *E. ictaluri*, however, it could not be mobilized to an *A. hydrophila* host, probably due to inability of *A. hydrophila* to recognize the plasmid origin of replication/transfer. *Edwardsiella* and *E. coli* are members of the *Enterobacteriaceae*, but *A. hydrophila* is member of family *Aeromonadaceae*. Therefore, the successful self-mobilization indicates that pEPMS-18199 is capable of transfer among *Enterobacteriaceae* members. In addition to *tra* regions, pEPMS-18199 also carries a DNA relaxase required for initiating plasmid DNA transfer during conjugation by cleaving a specific site at the origin of replication (Smillie et al., 2010). The results from conjugation experiment support the role of Tra regions in plasmid transfer by conjugation. Together, these data suggest the promotion of active transfer of pEPMS-18199 among bacterial strains (Cascales and Christie, 2003).

The plasmid stability experiment indicated that the *E. piscicida* host did not lose pEPMS-18199 following subculture without antibiotic pressure. pEPMS-18199 carries a partition system (*par*) involved in segregating plasmids to daughter cells during cell division (Baxter and Funnell, 2014; Brooks and Hwang, 2017). In fact, partition mechanisms are known to be responsible for the positioning of plasmids inside the cell as well as are the most important determinant for the stable maintenance of low-copy-number plasmids (Baxter and Funnell, 2014). Besides the partition system, the sequence of pEPMS-18199 revealed three plasmid addiction proteins, including restriction endonuclease subunit M, endonuclease, and DNA cytosine methyltransferase. Plasmid addiction systems help ensure that plasmids remain established in the bacterial population even in the absence of selection pressure by preventing the survival of plasmid-free cells due to selective killing (Tschäpe, 1994; Kroll et al., 2010). Plasmid partition proteins and addiction systems likely contribute to stable maintenance and persistence of pEPMS-18199 in host cells. The stability of pEPMS-18199 suggests that, in addition to providing the host with antibiotic resistance, this naturally occurring plasmid may also confer other advantages to the host under certain environment conditions.

To our knowledge, this study describes for the first time a high molecular weight conjugative plasmid carrying AMR genes in an *E. piscicida* isolate in the United States. The presence of *tetA/tetR, floR*, and *sul2* resistance genes on plasmid pEPMS-18199 mediate resistance to the three AMs approved for use in U.S. aquaculture. Interestingly, the frequency rate of MDR *E. piscicida* isolates is still low, resistant phenotypes was detected in 6.4% (3 out of 47) of the isolates among cases submitted to CVM/MSU Aquatic Research and Diagnostic Laboratory, Thad Cochran National Warmwater Aquaculture Center at Stoneville (unpublished data from 2018 annual case summary). Besides conferring the host strain with an AMR phenotype, pEPMS-18199 may also confer other advantages to the host. Careful and prudent use of antimicrobials in aquaculture production could help to reduce the persistence and propagation of such plasmid. Also, conducting AM sensitivity assays before antimicrobial therapy is strongly recommended.

## Data Availability Statement

The datasets generated for this manuscript can be found in GenBank under accession numbers CP035668.1 and CP035669.1

## Author Contributions

HA and ML designed the experiment. HA, RR, OO, and GW performed the experiment. HA wrote the draft. All authors read and review the manuscript.

### Conflict of Interest

The authors declare that the research was conducted in the absence of any commercial or financial relationships that could be construed as a potential conflict of interest.
