# Supplementary material for: Characterization of a Novel Conjugative Plasmid in Edwardsiella piscicida Strain MS-18-199
Source: Front Cell Infect Microbiol. 2019 Nov 27;9:404. doi: 10.3389/fcimb.2019.00404 (PMC6890552; doi:10.3389/fcimb.2019.00404)
Supplement: Supplementary file 1 [file Data_Sheet_1.docx]

**Supplementary table 1.**Results of biochemical tests used to identify *E. piscicida* strain MS-18-199.

| **Test** | **Results** |
| --- | --- |
| Lysine AMC | **+** |
| Xylose | **-** |
| Phosphate MU | **+** |
| Maltose | **-** |
| Proline AMC | **+** |
| Arabinose | **-** |
| γ -Glutamine 7AMC | **-** |
| Malonate | **-** |
| Ornithine | **+** |
| Sucrose | **-** |
| Bis-Phosphate | **-** |
| Inositol | **-** |
| Aesculin | **-** |
| Tryptophane Deaminase | **-** |
| FR6 | **-** |
| Citrate | **-** |
| Sorbitol | **-** |
| Beta-d-glucoside MU | **-** |
| Mannitol | **-** |
| Arabitol | **-** |
| Raffinose | **-** |
| Cellobiose | **-** |
| Agmatine | **+** |
| Urea | **-** |
| 2-acetamido-2-deoxyglucoside | **+** |
| Trehalose | **-** |
| Alpha-D-galactosideMU | **-** |
| Fructose | **+** |
| Lysine | **+** |
| Arginine | **-** |
| Pyruvate | **-** |
